# New Condensation Polymer Precursors Containing Consecutive Silicon Atoms—Decaisopropoxycyclopentasilane and Dodecaethoxyneopentasilane—And Their Sol–Gel Polymerization

**DOI:** 10.3390/polym11050841

**Published:** 2019-05-09

**Authors:** Sung Jin Park, Myong Euy Lee, Hyeon Mo Cho, Sangdeok Shim

**Affiliations:** 1 Department of Chemistry and Medical Chemistry, College of Science and Technology, Research and Education Center for Advanced Silicon Materials, Yonsei University, Wonju, Gangwon-do 26493, Korea; SungJin.Park@wacker.com (S.J.P.); melgg@yonsei.ac.kr (M.E.L.); 2 University College, Yonsei University, Incheon 21983, Korea; 3 Department of Chemistry, Sunchon National University, Sunchon 57922, Korea; san90@scnu.ac.kr

**Keywords:** sol–gel precursor, condensation polymer, alkoxycyclopentasilane, alkoxyneopentasilane, porosity, polysilane, polysiloxane, oxidation of Si–Si bond

## Abstract

The sol–gel polymerization of alkoxysilanes is a convenient and widely used method for the synthesis of silicon polymers and silicon–organic composites. The development of new sol–gel precursors is very important for obtaining new types of sol–gel products. New condensation polymer precursors containing consecutive silicon atoms—decaisopropoxycyclopentasilane (CPS) and dodecaethoxyneopentasilane (NPS)—were synthesized for the preparation of polysilane–polysiloxane material. The CPS and NPS xerogels were prepared by the sol–gel polymerization of CPS and NPS under three reaction conditions (acidic, basic and neutral). The CPS and NPS xerogels were characterized using N_2_ physisorption measurements (Brunauer–Emmett–Teller; BET and Brunauer-Joyner-Halenda; BJH), solid-state CP/MAS (cross-polarization/magic angle spinning) NMRs (nuclear magnetic resonances), TEM, and SEM. Their porosity and morphology were strongly affected by the structure of the precursors, and partial oxidative cleavage of Si-Si bonds occurred during the sol–gel process. The new condensation polymer precursors are expected to expand the choice of approaches for new polysilane–polysiloxane.

## 1. Introduction

Sol–gel polymerization of multialkoxysilanes is a convenient method for the synthesis of silicon polymers and silicon–organic composites under mild conditions [1,2,3,4,5,6,7,8,9]. A variety of sol–gel silicon polymer precursors such as alkyl or aryl bridged multialkoxysilane ((OR)_3_Si-R’-Si(OR)_3_, R’= alkyl, and aryl groups) and simple alkoxysilanes (Si(OR)_4_ and R’Si(OR)_3_) have been investigated [10,11,12,13]. Control of the morphologies and textural properties (surface area, pore volume, and pore distribution) of the resulting sol–gel polymer has been achieved by the molecular design of the precursors.

Quite recently, our group found interesting reactivities of peralkoxy-, perchlorocyclopentasilane, and perchloroneopentasilane: (i) Si–Si bond breaking of a soluble polymer synthesized from decaethoxycyclo*penta*silane with methyltrimethoxysilane occurred even at one minute-baking conditions (200 °C) [14], while a polymer obtained from hexaethoxyhexamethylcyclo*hexa*silane with methyltrimethoxysilane was thermally stable; (ii) adding a secondary amine into decachlorocyclopentasilane and dodecachloroneopentasilane [15] induced Si–Si bond cleavages [16,17,18,19,20]. The interesting cleavage reactivity of the consecutive silicon bonds in the oligosilanes and the lack of study on the sol–gel process of peralkoxyoligosilanes prompted us to study their sol–gel reaction. To the best of our knowledge, sol–gel reactions of oligosilanes have not been studied. Only commercially available simple alkoxydisilanes—(OEt)_3_SiSi(OEt)_3_ and (OMe)_3_SiSi(OMe)_3_—were used for the synthesis of sol–gel silicon oxide materials [21,22]. We expected that the new core structures and Si–Si bond breaking may affect the morphology and textural property of the resulting sol–gel polymer.

In this report, we describe the synthesis of new condensation polymer precursors containing consecutive silicon atoms—decaisopropoxycyclopentasilane (CPS) and dodecaethoxyneopentasilane (NPS)—as well as their sol–gel reactions and structures of the resulting CPS and NPS xerogels, representing the first sol–gel study of peralkoxyoligosilane.


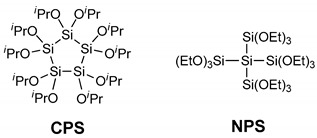


## 2. Materials and Methods

### 2.1. Materials

In all of the reactions in which air-sensitive chemicals were used, the reagents and solvents were dried prior to use. Diethyl ether, triethylamine, and *n*-hexane were distilled from Na/Ph_2_CO. EtOH and *^i^*PrOH were distilled from calcium hydride. Other starting materials were purchased as reagent grade and were used without further purification. Glassware was flame-dried with nitrogen or argon flushing prior to use. All of the manipulations were performed using the standard Schlenk techniques in nitrogen or argon atmosphere and using a glove box (MBraun, Garching, Germany).

### 2.2. Instruments and Measurements

^1^H, ^13^C, and ^29^Si NMR spectra were recorded using a Bruker Avance II^+^ BBO 400 MHz S1 spectrometer (Bruker, Billerica, MA, USA). The chemical shifts were referenced to internal C_6_D_6_ or CDCl_3_, or external tetramethylsilane. All of the solid-state NMR experiments were conducted with NMR instruments (Varian ^unity^NOVA, Varian, Palo Alto, CA, USA) using 5 and 2.5 mm double resonance MAS probe heads for ^29^Si and ^1^H at 14.1 T (^1^H resonance frequency 600 MHz, wide bore) at room temperature. Mass spectra were recorded using a low-resolution (Agilent Technologies GC/MS: 6890N, 5973N mass selective detector) EI (electron ionization) mass spectrometer and a high-resolution (JEOL JMS-600W Agilent 6890 Series, Agilent, Santa Clara, CA, USA) instrument. The morphology of the xerogels was monitored by scanning electron microscopy (SEM, Quanta 250FEG, FEI, Hillsboro, OR, USA). High-resolution transmission electron microscopy (HRTEM) was performed with a JEOL JEM2100F (200 kV) instrument (JEOL, Tokyo, Japan) using a carbon-coated 200-mesh copper grid. Nitrogen adsorption–desorption isotherms were obtained using a surface area analyzer (BELSORP-max and Micromeritics, ASAP 2010, BEL, Osaka, Japan). Thermogravimetric analysis (TGA) curves were recorded using a simultaneous thermal analyzer (STA, STA 8000, Perkin Elmer, Waltham, MA, USA) in N_2_ (50.0 mL/min) atmosphere with heating from 30.0 to 1000.0 °C (10.0 °C/min).

### 2.3. Synthesis of Decaisopropoxycyclopentasilane (CPS, Si_5_(O^i^Pr)_10_)

*^i^*PrOH (5.8 mL, 0.076 mol) and Et_3_N (10.6 mL, 0.076 mol) dissolved in Et_2_O (150 mL) were slowly added to decachlorocyclopentasilane [23,24] (3.1 g, 6.3 mmol) dissolved in Et_2_O (350 mL) for 1 h at −78 °C. After the mixture was stirred for 6 h at −78 °C and slowly warmed to room temperature, the solution was stirred for 12 h at room temperature. The Et_3_N^+^Cl^−^ salt was removed by filtration and washing with *n*-hexane in argon atmosphere in a glove box, and volatiles were distilled using vacuum distillation. Solid CPS (4.0 g, 5.5 mmol) was obtained at 88% yield. Si_5_(O*^i^*Pr)_10_ (CPS): ^1^H NMR (C_6_D_6_, 400 MHz): *δ* 4.58 (m, 10H, SiO*CH*(CH_3_)_2_), 1.40 (d, 60H, SiO*C*H(*CH_3_*)_2_). ^13^C NMR (C_6_D_6_, 100 MHz): *δ* 26.79 (s, SiO*C*H(*CH_3_*)_2_), 69.17 (s, SiO*CH*(CH_3_)_2_). ^29^Si NMR (C_6_D_6_, 79 MHz): *δ* −14.41 (s). HRMS (high resolution mass): C_27_H_63_O_10_Si_5_ 687.3268 (M^+^-*^i^*Pr calcd), 687.3267 (found).

### 2.4. Synthesis of Dodecaethoxyneopentasilane (NPS, Si_5_(OEt)_12_)

EtOH (3.2 mL, 0.055 mol) and Et_3_N (8.4 mL, 0.060 mol) dissolved in Et_2_O (150 mL) were slowly added to the dodecaethoxyneopentasilane [25] (3.1 g, 6.3 mmol) dissolved in Et_2_O (350 mL) for 1 h at −78 °C. After the mixture was stirred for 6 h at −78 °C and slowly warmed to room temperature, the solution was stirred for 12 h at room temperature. The Et_3_N^+^Cl^−^ salt was removed by filtration and washing with *n*-hexane in argon atmosphere in a glove box, and volatiles were distilled using vacuum distillation. NPS (2.3 g, 3.4 mmol) was obtained at 82% yield as an oil. Si_5_(OEt)_12_ (NPS): ^1^H NMR (C_6_D_6_, 400 MHz): *δ* 3.87 (q, 24H, SiO*CH_2_*CH_3_), 1.16 (t, 36H, SiOCH_2_*CH_3_*). ^13^C NMR (C_6_D_6_, 100 MHz): *δ* 18.90 (s, SiOCH_2_*CH_3_*), 58.85 (s, SiO*CH_2_*CH_3_). ^29^Si NMR (C_6_D_6_, 79 MHz): *δ* −40.52 (s, Si(*Si*(OEt)_3_)*_4_*), −152.16 (s, *Si*(Si(OEt)_3_)*_4_*). HRMS: C_24_H_61_O_12_Si_5_ 681.3009 (M^+^ calcd), 681.3030 (found).

### 2.5. Sol–Gel Polymerization of CPS and NPS

The sol–gel polymerization of CPS and NPS were carried out at 0.4 M concentration in EtOH or *^i^*PrOH according to the substituent groups of the precursors, respectively. The monomers were dissolved in anhydrous alcohols in 25.0 mL vials. An aqueous catalyst (0.01 wt % HCl or NaOH per water) was added to the vial. The solutions were sealed and shaken at room temperature. Gelation was determined at a point where the solution did not flow as a liquid. After gelation, the monoliths were aged for 2 weeks at 30 °C prior to further processing. The gels were crushed in water (100 mL), and filtered and washed with water (2 × 100 mL) and alcohols (EtOH or *^i^*PrOH, 2 × 100 mL). After drying overnight in air, the xerogels were prepared by vacuum drying at 50 °C for 24 h.

### 2.6. Preparation of CPSH

CPS (1.7 g, 2.32 mmol) was diluted in anhydrous *^i^*PrOH (4.5 mL) to adjust to 0.4 M concentration in an oven-dried vial (25 mL). A prepared aqueous acid catalyst, namely, 0.01 wt % HCl dissolved in 10 eq of H_2_O (0.42 mL, 23 mmol), was added to the vial, and then the vial was sealed and shaken. Gelation occurred within 5 min. After aging for 2 weeks at 30 °C, the wet gel was crushed in distilled H_2_O (100 mL) and filtered. The solids were washed twice with distilled H_2_O (100 mL) and anhydrous *^i^*PrOH (100 mL). After drying for 12 h in air, a white solid CPSH xerogel (0.54 g) was obtained by vacuum drying for 24 h at 50 °C. CPSH: ^1^H CP/MAS NMR (600 MHz): *δ* 0.975, 1.138, 5.126. ^13^C CP/MAS NMR (150 MHz): *δ* −1.862, 23.385, 47.661, 65.787. ^29^Si CP/MAS NMR (120 MHz): *δ* −29.062 (D), −70.335 (T), −100.860 (Q). N_2_ physisorption surface area (Brunauer–Emmett–Teller; BET) [26,27]: 317 m^2^/g, N_2_ absorption mean pore diameter (Brunauer-Joyner-Halenda; BJH): 28, 50, 90 nm. Total weight loss (TGA, 50–1000 °C, N_2_ atmosphere): 19.3%.

### 2.7. Preparation of CPSOH

Similarly to the procedure for the preparation of CPSH, CPSOH was prepared using an aqueous base catalyst, namely, 0.01 wt % NaOH dissolved in 10 eq of H_2_O (0.42 mL, 23 mmol), instead of the acid catalyst. Gelation occurred within 3 min. After aging, washing and drying were carried out in the same manner as described above. A white solid CPSOH xerogel (0.51 g) was obtained. CPSOH: ^1^H CP/MAS NMR (600 MHz): *δ* 0.690, 0.975, 1.138, 5.329. ^13^C CP/MAS NMR (150 MHz): *δ* 22.738, 65.657. ^29^Si CP/MAS NMR (120 MHz): *δ* −26.175 (D), −69.782 (T), −101.229 (Q). N_2_ physisorption surface area (BET): 408 m^2^/g, N_2_ absorption mean pore diameter (BJH): 24, 43, 76 nm. Total weight loss (TGA, 50–1000 °C, N_2_ atmosphere): 17.8%.

### 2.8. Preparation of CPSN

Similarly to the procedure used for the preparation of CPSH, CPSN was prepared using 10 eq of distilled H_2_O (0.42 mL, 23 mmol) without any acid or base catalyst. Gelation occurred within 10 min. After aging, washing and drying were carried out in the same manner as described above. A white solid CPSN xerogel (0.53 g) was obtained. CPSN: ^1^H CP/MAS NMR (600 MHz): *δ* 0.120, 0.527, 4.190. ^13^C CP/MAS NMR (150 MHz): *δ* 0.534, 24.615, 30.182, 65.978. ^29^Si CP/MAS NMR (120 MHz): *δ* −32.076 (D), −71.382 (T), −100.984 (Q). N_2_ physisorption surface area (BET): 150 m^2^/g, N_2_ absorption mean pore diameter (BJH): 32, 50, 76 nm. Total weight loss (TGA, 50–1000 °C, N_2_ atmosphere): 16.2%.

### 2.9. Preparation of NPSH

The NPS (1.8 g, 2.6 mmol) was diluted in anhydrous EtOH (4.3 mL) to adjust to 0.4 M concentration in an oven-dried vial (25 mL). A prepared aqueous acid catalyst, namely, 0.01 wt % HCl dissolved in 12 eq of H_2_O (0.56 mL, 31 mmol), was added to the vial and then the vial was sealed and shaken. Gelation occurred within 4 days. After aging for 2 weeks at 30 °C, the wet gel was crushed in distilled H_2_O (100 mL) and filtered. The solids were washed twice with distilled H_2_O (100 mL) and anhydrous EtOH (100 mL). After drying for 12 h in air, a white solid NPSH xerogel (0.63 g) was obtained by vacuum drying for 24 h at 50 °C. NPSH: ^1^H CP/MAS NMR (600 MHz): *δ* 0.080, 0.487, 3.905. ^13^C CP/MAS NMR (150 MHz): *δ* 1.052, 17.688, 29.987, 59.117. ^29^Si CP/MAS NMR (120 MHz): *δ* −80.472 (T), −101.721, −109.951 (Q). Total weight loss (TGA, 50–1000 °C, N_2_ atmosphere): 14.9%.

### 2.10. Preparation of NPSOH

Similarly to the procedure used for the preparation of NPSH, NPSOH was prepared using an aqueous base catalyst, namely, 0.01 wt % NaOH dissolved in 12 eq of H_2_O (0.56 mL, 31 mmol), instead of the acid catalyst. Gelation occurred within 1 day. After aging, washing and drying were carried out in the same manner as described above. A white solid NPSOH xerogel (0.67 g) was obtained. NPSOH: ^1^H CP/MAS NMR (600 MHz): *δ* 0.202, 2.806, 6.306. ^13^C CP/MAS NMR (150 MHz): *δ* 17.365, 30.117, 59.894. ^29^Si CP/MAS NMR (120 MHz): *δ* −91.158 (T), −101.721, −110.688 (Q). N_2_ physisorption surface area (BET): 7 m^2^/g, N_2_ absorption mean pore diameter (BJH): 66, 90, 119 nm. Total weight loss (TGA, 50–1000 °C, N_2_ atmosphere): 27.4%.

### 2.11. Preparation of NPSN

Similarly to the procedure for the preparation of NPSH, NPSOH was prepared using 12 eq of distilled H_2_O (0.56 mL, 31.0 mmol) without any acid or base catalyst. Gelation occurred within 2 days. After aging, washing and drying were carried out in the same manner as described above. A white solid NPSN xerogel (0.62 g) was obtained. NPSN: ^1^H CP/MAS NMR (600 MHz): *δ* 0.527, 3.986. ^13^C CP/MAS NMR (150 MHz): *δ* 1.700, 17.753, 30.052, 58.793. ^29^Si CP/MAS NMR (120 MHz): *δ* −80.717 (T), −101.844, −110.442 (Q). N_2_ physisorption surface area (BET): 28 m^2^/g, N_2_ absorption mean pore diameter (BJH): 21, 43, 120 nm. Total weight loss (TGA, 50–1000 °C, N_2_ atmosphere): 17.9%.

## 3. Results and Discussion

### 3.1. Synthesis of the New Condensation Polymer Precursors

**Scheme 1:**
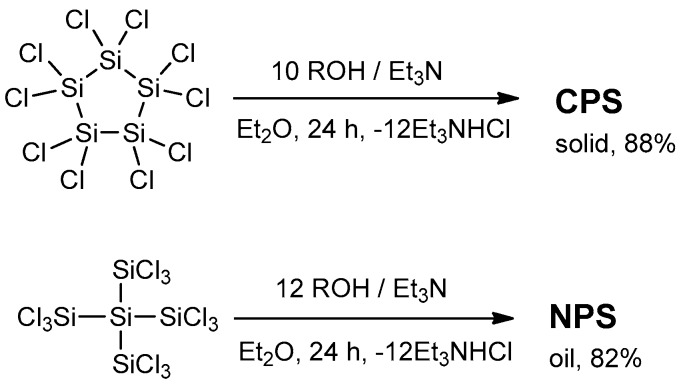
Synthesis of the new condensation polymer precursors—decaisopropoxycyclopentasilane (CPS) and dodecaethoxyneopentasilane (NPS).

To investigate the sol–gel reaction of the oligosilane with consecutive silicon bonds by the molecular designed precursors, cyclic and branched peralkoxyoligosilanes were synthesized. The decaisopropoxycyclopentasilane (CPS)—Si_5_(O^i^Pr)_10_—and dodecaethoxyneopentasilane (NPS)—Si(Si(OEt)_3_)_4_—were synthesized by the alcoholysis of decachlorocyclopentasilane and dodecachloroneopentasilane, respectively (Scheme 1). The alcoholysis reactions were carried out at −78 °C in Et_2_O for 6 h, and then the reaction mixture was slowly warmed to room temperature. After 12 h of stirring at room temperature, the formed white salt (Et_3_N^+^Cl^−^) was removed by filtration without contact with air and moisture. The CPS cyclic compound was obtained as a solid at 88% yield. Using a method similar to the method described above, branched precursor NPS was obtained as an oil at 82% yield. The two products were characterized by ^1^H NMR (Figures S1 and S4), ^13^C NMR, ^29^Si NMR (Figures S2 and S5), and HRMS (Figures S3 and S6). The ^29^Si NMR spectrum of CPS displayed a resonance at −14.4 ppm. In the HRMS data, one ^i^Pr group removed fragment of CPS was detected. Two resonances were observed at −40.5 and −152.2 ppm in the ^29^Si NMR spectrum of NPS and were assigned to the four terminal Si atoms and the central Si atom, respectively.

### 3.2. Preparation of CPS and NPS Xerogels

**Scheme 2:**
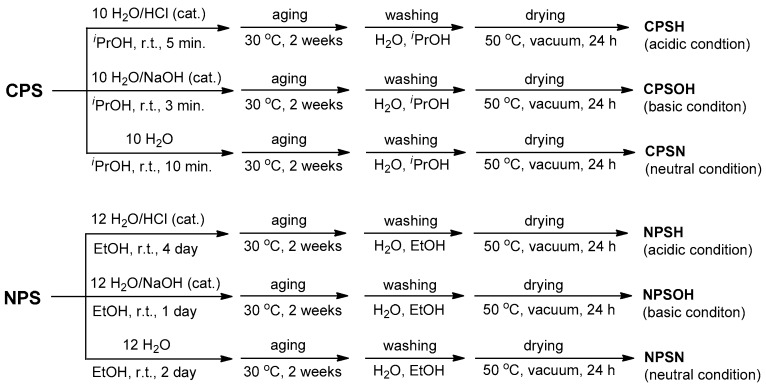
Preparation of CPS and NPS xerogels.

To prepare polysilane–polysiloxane materials, sol–gel polymerizations of CPS and NPS (0.4 M) were carried out at room temperature in isopropanol or ethanol according to the substituent groups of the precursors. Hydrolysis and condensation reactions were carried out in neutral, acidic (0.01 wt % HCl per H_2_O), and basic (0.01 wt % NaOH per H_2_O) conditions. For the hydrolysis, water was used with the number of moles of the equivalent of alkoxy groups of each precursor (10 eq for CPS and 12 eq for NPS). The CPS solution became a viscous gel within a few minutes, while the gelation of NPS required a few days. After gelation, all of the gels were aged for 2 weeks at room temperature to obtain condensation that was as complete as possible prior to further processing. The wet gels were washed with water and the corresponding solvent to remove the catalyst. Xerogels were obtained as opaque white brittle solids by vacuum drying for 24 h at 50 °C. The xerogels were ground into fine powder for analyses (Scheme 2).

### 3.3. Characterization of CPS and NPS Xerogels

#### 3.3.1. Surface Area and Porosity

The porosity of the xerogels was determined by N_2_ physisorption measurements. The textural properties of xerogels are summarized in Table 1. The BET (Brunauer–Emmett–Teller) method was applied to determine the surface area of the xerogels (Figures S7 and S8). CPS xerogels showed a larger surface area than the NPS xerogels. CPSOH exhibited the largest surface area of 408 m^2^·g^−1^ and a total pore volume (at *P*/*P_0_* = 0.99) of 0.74 cm^3^·g^−1^. CPSH showed a quite large surface area of 317 m^2^·g^−1^ and the largest total pore volume of 0.83 cm^3^·g^−1^. By contrast, the NPS xerogels were either non-porous or exhibited negligible porosity. These results indicated that the designed structure of the precursors affected the surface area and the porosity of the xerogels. Considering that the core size of CPS is larger than that of NPS, it was assumed that the higher porosity of CPS may be due to the difference in the core size. Usually, bridged polysilsequioxanes, which are large-core silsesquixoanes, are highly porous and have high surface areas [10,11,12,28,29,30,31,32].

#### 3.3.2. Solid-State CP/MAS NMR Analysis

**Figure 1:**
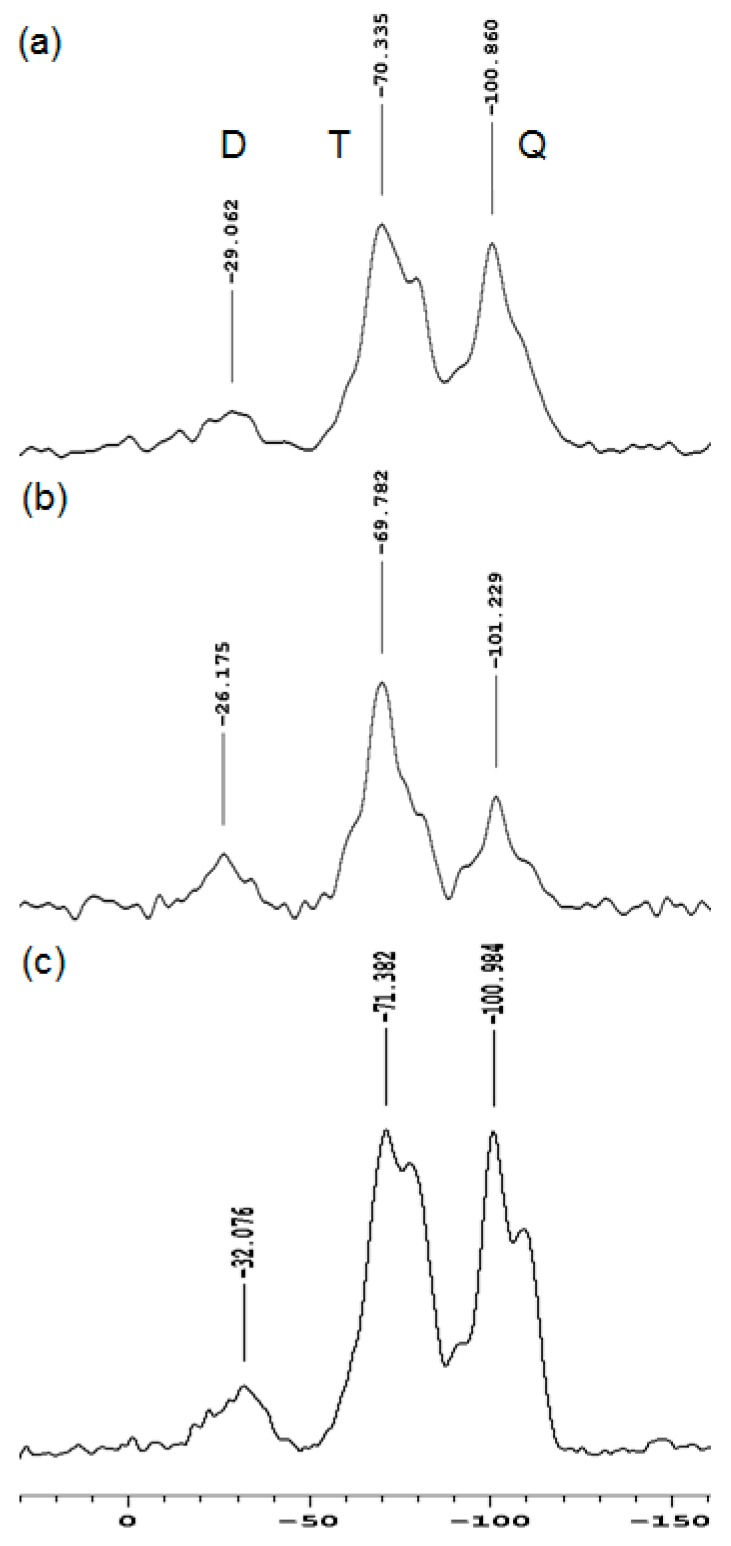
^29^Si CP/MAS NMR spectra of CPS xerogels. (**a**) CPSH, (**b**) CPSOH, (**c**) CPSN.

**Figure 2:**
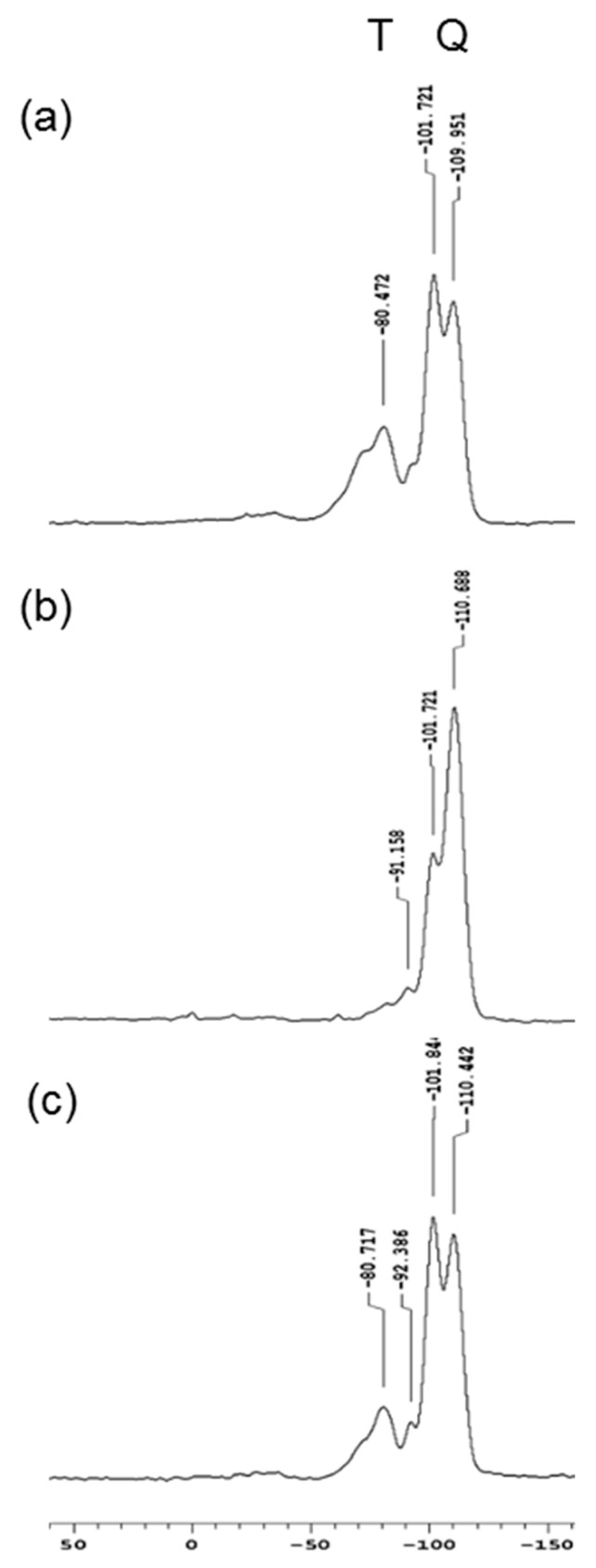
^29^Si CP/MAS NMR spectra of NPS xerogels. (**a**) NPSH, (**b**) NPSOH, (**c**) NPSN.

The xerogels were characterized by solid-state ^1^H, ^13^C, and ^29^Si CP/MAS NMR spectroscopy. Shorthand notations have been commonly used in the organosilicon literature for the Si–O bonding configurations: The structures of (SiO)_1_Si, (SiO)_2_Si, (SiO)_3_Si, and (SiO)_4_Si are referred to as M, D, T, and Q, as this relates to the number of O atoms bonded to a Si atom, respectively. ^29^Si CP/MAS NMR spectra of the CPS xerogels show the presence of D (R_2_SiO_2_) (*δ* from −20 to −40 ppm), T (RSiO_3_) (*δ* from −60 to −80 ppm), and Q (SiO_4_) units (*δ* from −90 to −120 ppm) [33,34,35,36,37]. If there was no Si–Si bond cleavage of CPS during the sol–gel process, only the D unit should be observed. However, T and even Q units were observed in Figure 1. The spectra indicate that the oxidative cleavage of Si-Si bonds in cyclopentasilane partially occurred during the sol–gel reaction to give Si–O–Si bonds [38,39,40,41,42,43,44]. Generally, when materials with Si–Si bonds on the surface are immersed into water or exposed to dry air, the Si–Si bonds are readily oxidized, and chemically stabilized Si–O–Si bonds are formed. The surface Si–Si bonds of the xerogels may be oxidized since NPS and CPS were exposed to water and air for a long time. Similarly to the ^29^Si CP/MAS NMR spectrum of CPS xerogels, the Q unit is also found in the spectra of NPS xerogels. Without Si–Si bond breaking of NPS during the sol–gel process, only the T unit should be observed. The resonances at the T and Q units of NPS xerogels observed in Figure 2 imply the partial oxidation of the Si–Si bonds of neopentasilane.

#### 3.3.3. Microscopy

**Figure 3:**
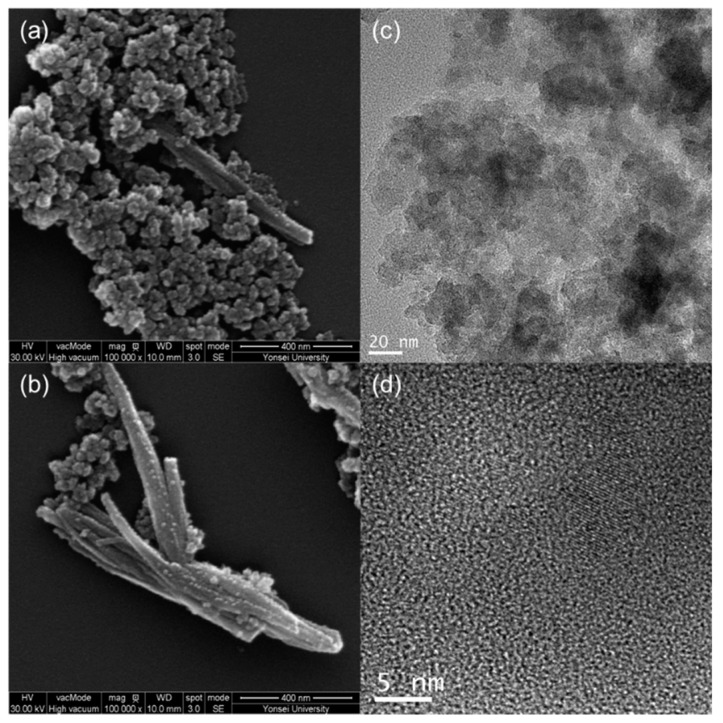
Morphology and pore texture of CPSH. SEM images (**a**,**b**) and TEM images (**c**,**d**).

**Figure 4:**
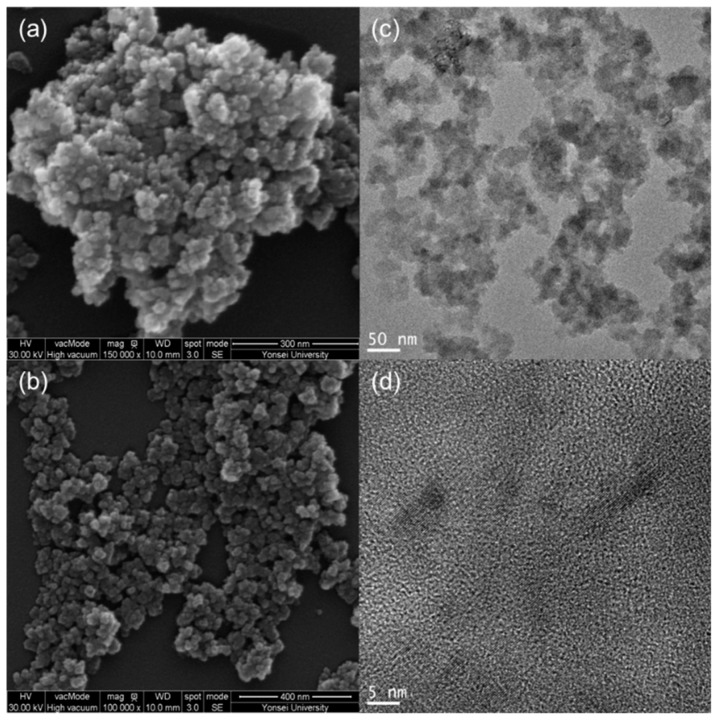
Morphology and pore texture of CPSOH. SEM images (**a**,**b**) and TEM images (**c**,**d**).

**Figure 5:**
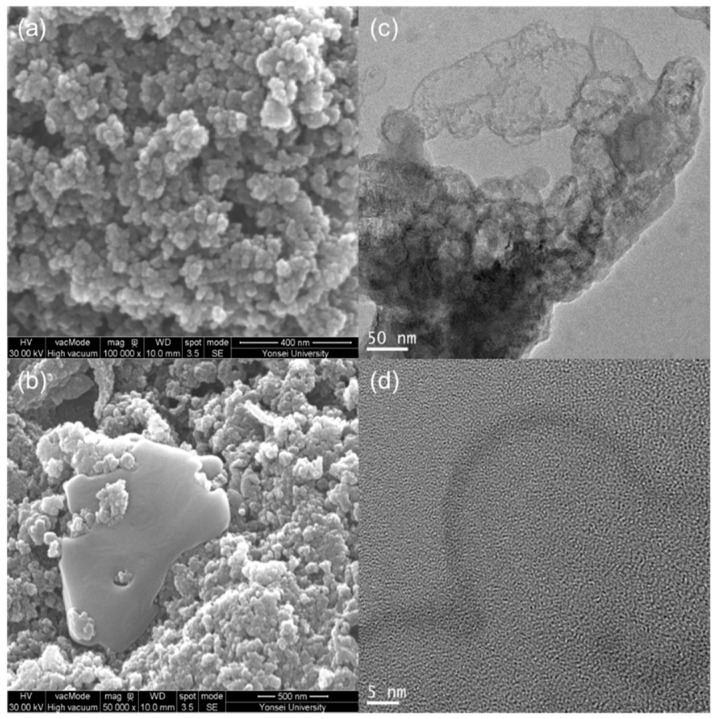
Morphology and pore texture of CPSN. SEM images (**a**,**b**) and TEM images (**c**,**d**).

The morphology and pore texture of xerogels were investigated by scanning electron microscopy (SEM) and high-resolution transmission electron microscopy (HRTEM). For CPS xerogels, Figure 3, Figure 4 and Figure 5 show disordered meso- and macroporous structures consisting of spherical nanoparticles with other shapes. The sizes of the spherical nanoparticles were measured as 10–20 nm (CPSH), 20–30 nm (CPSOH), and 30–90 nm (CPSN). The different sizes of the particles can be attributed to the different conditions of the sol–gel reactions. While CPSOH consisted of spherical nanoparticles only (Figure 4a,b), aggregated nanorods sized 400–500 nm in CPSH (Figure 3b) and a huge particle sized approximately 1.5 µm in CPSN (Figure 5b) were observed along with spherical nanoparticles. These noncrystalline composites may have contributed to the reduction of the surface areas of CPSH and CPSN (Table 1). TEM images display the mesoporous textures of the CPS xerogels (Figure 3c,d, Figure 4c,d, and Figure 5c,d).

**Figure 6:**
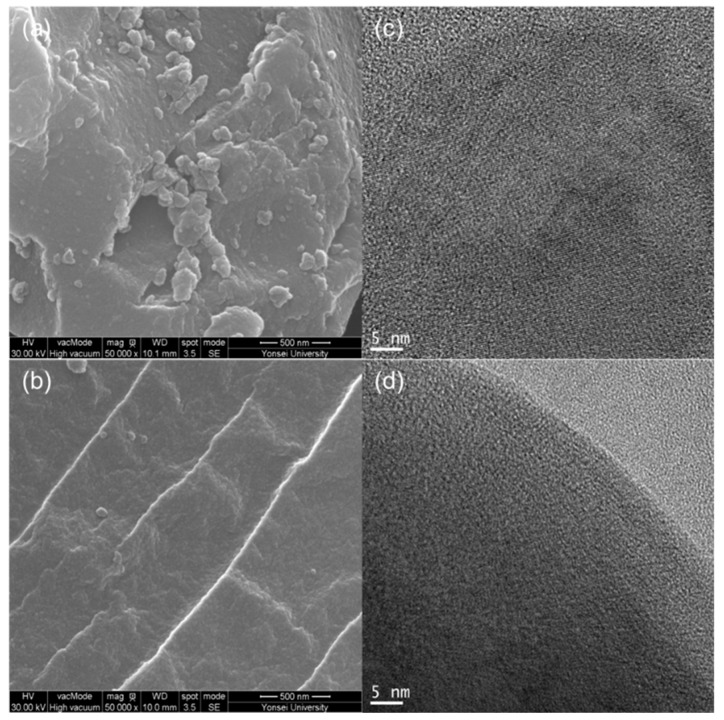
Morphology of NPSH. SEM images (**a**,**b**) and TEM images (**c**,**d**).

**Figure 7:**
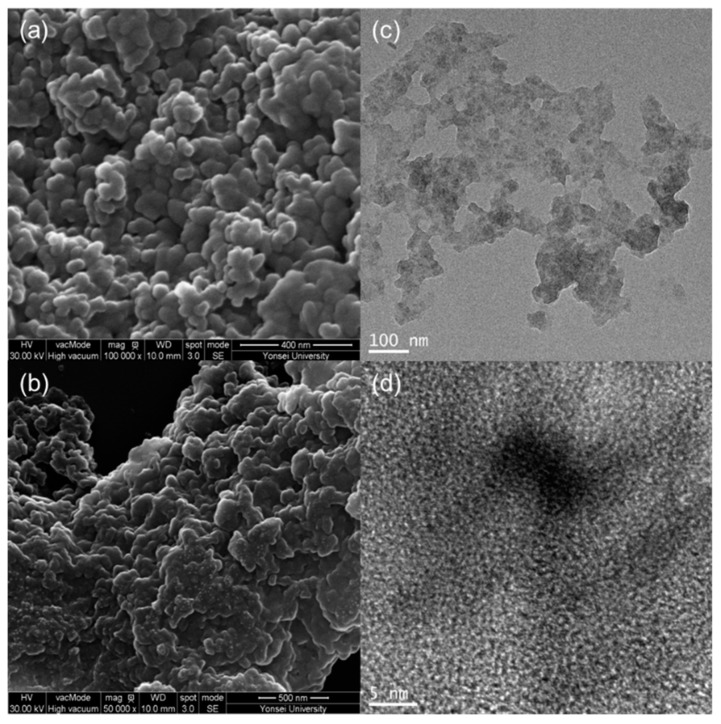
Morphology of NPSOH. SEM images (**a**,**b**) and TEM images (**c**,**d**).

**Figure 8:**
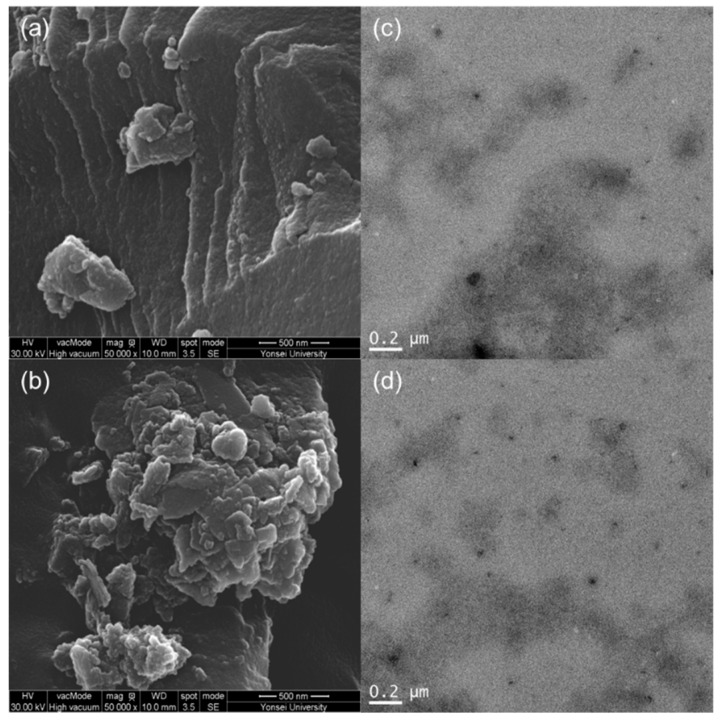
Morphology of NPSN. SEM images (**a**,**b**) and TEM images (**c**,**d**).

The morphologies of NPS xerogels were observed to be significantly different from those of the CPS xerogels. NPSH and NPSN are flat plates connected to each other as stacked films, and NPSOH is an irregularly wrinkled plate. In the TEM images, the textures consist of highly crystalline composites with no pores (Figure 6, Figure 7 and Figure 8).

## 4. Conclusions

The new condensation polymer precursors containing consecutive silicon atoms—CPS and NPS—were successfully synthesized at high yields. CPS and NPS xerogels were prepared by sol–gel polymerization under three reaction conditions (acidic, basic, and neutral) using the new precursors. The characterizations of CPS and NPS xerogels using BET, BJH, and microscopy showed that their porosity and morphology were strongly affected by the structure of precursors. The CPS xerogels exhibited relatively large surface areas and total pore volumes, while the NPS xerogels exhibited nonporosity or negligible porosity. The relatively high porosity of CPS xerogels was achieved without any organic substituent, template, or porogen, and they showed 16~19% of weight loss in TGA analysis. Bridged polysilsesquioxanes exhibited higher porosity, however, the weight loss reached around 45% [10,29]. ^29^Si CP/MAS NMR study of the xerogels indicated that a partial oxidative cleavage of the Si–Si bonds in cyclopentasilane and neopentasilane occurred during the sol–gel process. The structural features—a hybrid of Si–Si and Si–O–Si bonds—might provide a new optical property since consecutive silicon bonds absorb UV-visible light [14], and porous silica shows photoluminescence. The mixing of different skeletal bonds—Si–O–Si in an insulator and Si-Si in a semiconductor—provided interesting thermal and electrical conductivity. We hope that the new condensation polymer precursors for polysilane–polysiloxane materials will be used in many applications such as core-shell particle, anode materials for Li-ion batteries and non-metal thermal conductors.

## Figures and Tables

**Table 1:** Textural properties of the xerogels (BET and BJH).

Xerogels	*Area S* (m^2^/g)	*Pore Volume* (cm^3^/g)	*Mean Pore Diameter* (nm)
CPSH	317	0.83	28, 50, 90
CPSOH	408	0.74	24, 43, 76
CPSN	150	0.24	32, 50, 76
NPSH	No porosity	-	-
NPSOH	7	0.02	66, 90, 119
NPSN	28	0.06	21, 43, 120

## Supplementary Materials

The following are available online at https://www.mdpi.com/2073-4360/11/5/841/s1, Figure S1: ^1^H NMR spectrum of CPS, Figure S2: ^29^Si NMR spectrum of CPS, Figure S3: HRMS result of CPS, Figure S4: ^1^H NMR spectrum of NPS, Figure S5: ^29^Si NMR spectrum of NPS, Figure S6: HRMS result of NPS, Figure S7: N_2_ adsorption–desorption isotherms of the CPS xerogels. (a) CPSH, (b) CPSOH, (c) CPSN. Figure S8: Pore size distribution of the CPS xerogels. (a) CPSH, (b) CPSOH, (c) CPSN.

## References

[1] Brinker C.J., Scherer G.W. (1990). Introduction. Sol-Gel Science: The Physics and Chemistry of Sol-Gel Processing.

[2] Brinker C.J., Scherer G.W. (1990). Hydrolysis and Condensation II: Silicates. Sol-Gel Science: The Physics and Chemistry of Sol-Gel Processing.

[3] Brook M.A. (2000). Siloxane based on T and Q units. Silicon in Organic, Organometallic, and Polymer Chemistry.

[4] Wilkes G.L., Huang H.-H., Glaser R.H., Ziegler J.M., Fearon F.W.G. (1990). New Inorganic-Organic Hybrid Materials Through Sol-Gel Approach. Silicon-Based Polymer Science.

[5] Avnir D., Klein L.C., Levy D., Schubert U., Wojcik A.B., Rappoport Z., Apeloig Y. (1998). Organo-silica sol-gel materials. The Chemistry of Organic Silicon Compounds.

[6] Hench L.L., West J.K. (1990). The Sol-Gel Process. Chem. Rev..

[7] Schottner G. (2001). Hybrid Sol-Gel-Derived Polymers: Applications of Multifunctional Materials. Chem. Mater..

[8] Ciriminna R., Fidalgo A., Pandarus V., Béland F., Ilharco L.M., Pagliaro M. (2013). The Sol–Gel Route to Advanced Silica-Based Materials and Recent Applications. Chem. Rev..

[9] Danks A.E., Hall S.R., Schnepp Z. (2016). The evolution of ‘sol–gel’ chemistry as a technique for materials synthesis. Mater. Horiz..

[10] Loy D.A., Shea K.J. (1995). Bridged Polysilsesquioxanes. Highly Porous Hybrid Organic-Inorganic Materials. Chem. Rev..

[11] Croissant J.G., Cattoën X., Wong Chi Man M., Durand J.-O., Khashab N.M. (2015). Syntheses and applications of periodic mesoporous organosilica nanoparticles. Nanoscale.

[12] Shea K.J., Loy D.A. (2001). Bridged Polysilsesquioxanes. Molecular-Engineered Hybrid Organic-Inorganic Materials. Chem. Mater..

[13] Baney R.H., Itoh M., Sakakibara A., Suzuki T. (1995). Silsesquioxanes. Chem. Rev..

[14] Park S.J., Cho H.M., Lee M.E., Kim M., Han G., Hong S., Lim S., Lee H., Hwang B., Kim S.K. (2015). Soluble polycyclosilane–polysiloxane hybrid material and silicon thin film with optical properties at 193 nm and etch selectivity. J. Mater. Chem. C.

[15] Park S.J., Choi J.M., Cho H.M., Kim C.H., Lee M.E. (2014). *N*-Methylaniline-induced Si-Si bond cleavages of perchlorooligosilanes. J. Organomet. Chem..

[16] Wilkins C.J. (1953). The Reductions of Hexachlorodisilane with Ammonium Halides and Trimethylamine Hydrohalides. J. Chem. Soc..

[17] Kummer D., Köster H. (1971). 1,4-Dihydro-1,4-bis-(trichlorosilyl)pyridine from Si_2_Cl_6_ and Pyridine. Angew. Chem. Int. Ed. Engl..

[18] Tillmann J., Meyer-Wegner F., Nadj A., Becker-Baldus J., Sinke T., Bolte M., Holthausen M.C., Wagner M., Lerner H.-W. (2012). Unexpected Disproportionation of Tetramethylethylenediamine-Supported Perchlorodisilane Cl_3_SiSiCl_3_. Inorg. Chem..

[19] Zhang J., Xie J., Lee M.E., Zhang L., Zuo Y., Feng S. (2016). Ionic S_N_i-Si Nucleophilic Substitution in *N*-Methylaniline-Induced Si-Si Bond Cleavages of Si_2_Cl_6_. Chem. Eur. J..

[20] Schweizer J.I., Meyer L., Nadj A., Diefenbach M., Holthausen M.C. (2016). Unraveling the Amine-Induced Disproportionation Reaction of Perchlorinated Silanes—A DFT Study. Chem. Eur. J..

[21] Fernández-Sánchez C., Rodríguez J.A., Domínguez C. (2015). Synthesis of sol-gel SiO_2_-based materials using alkoxydisilane precursors: mechanisms and luminescence studies. J. Sol-Gel Sci. Technol..

[22] Rodríguez J.A., Fernández-Sánchez C., Domínguez C., Hernández S., Berencén Y. (2012). Bulk silica-based luminescent materials by sol–gel processing of non-conventional precursors. Appl. Phys. Lett..

[23] Hengge E., Kovar D. (1977). Zur reaktion der silicium—Phenyl-bindung mit HCl. J. Organomet. Chem..

[24] Kovar D., Utvary K., Hengge E. (1979). ^29^Si-NMR-Untersuchungen an einigen Cyclosilanderivaten. Monatsh. Chem..

[25] Cannady J.P., Zhou X. (2008). Composition Comprising Neopentasilane and Method of Preparing Same. WO.

[26] Brunauer S., Emmett P.H., Teller E.J. (1938). Adsorption of Gases in Multimolecular Layers. J. Am. Chem. Soc..

[27] Langmuir I. (1918). The adsorption of gases on plane surfaces of glass, mica and platinum. J. Am. Chem. Soc..

[28] Oviatt H.W., Shea K.J., Small J.H. (1993). Alkylene-bridged silsesquioxane sol-gel synthesis and xerogel characterization. Molecular requirements for porosity. Chem. Mater..

[29] Small J.H., Shea K.J., Loy D.A. (1993). Arylene- and alkylene-bridged polysilsesquioxanes. J. Non-Cryst. Solids.

[30] Shea K.J., Loy D.A., Webster O. (1992). Arylsilsesquioxane gels and related materials. New hybrids of organic and inorganic networks. J. Am. Chem. Soc..

[31] Barrie P.J., Carr S.W., Ou D.L., Sullivan A.C. (1995). Formation and Characterization of Novel Organodisilicate Glasses. Chem. Mater..

[32] Birault A., Molina E., Toquer G., Lacroix-Desmazes P., Marcotte N., Carcel C., Katouli M., Bartlett J.R., Gérardin C., Wong Chi Man M. (2018). Large-Pore Periodic Mesoporous Organosilicas as Advanced Bactericide Platforms. ACS Appl. Biol. Mater..

[33] Engelhardt G., Jancke H., Mägi M., Pehk T., Lippmaa E. (1971). Über die ^1^H-, ^13^C- und ^29^Si-NMR chemischen Verschiebungen einiger linearer, verzweigter und cyclischer Methylsiloxan-Verbindungen. J. Organomet. Chem..

[34] Williams E.A., Patai S., Rappoport Z. (1989). NMR spectroscopy of organosilicon compounds. The Chemistry of Organic Silicon Compounds.

[35] Mabboux P.-Y., Gleason K.K. (2005). Chemical Bonding Structure of Low Dielectric Constant Si:O:C:H Films Characterized by Solid-State NMR. J. Electrochem. Soc..

[36] Fyfe C.A., Zhang Y., Aroca P. (1992). An alternative preparation of organofunctionalized silica gels and their characterization by two-dimensional high-resolution solid-state heteronuclear NMR correlation spectroscopy. J. Am. Chem. Soc..

[37] Kuo A.C.M., Mark J.E. (1999). Poly(dimethylsiloxane). Polymer Data Handbook.

[38] Yang D.-Q., Gillet J.-N., Meunier M., Sacher E. (2005). Room temperature oxidation kinetics of Si nanoparticles in air, determined by x-ray photoelectron spectroscopy. J. Appl. Phys..

[39] Wolkin M.V., Jorne J., Fauchet P.M., Allan G., Delerue C. (1999). Electronic States and Luminescence in Porous Silicon Quantum Dots: The Role of Oxygen. Phys. Rev. Lett..

[40] Ritter J.C., Robinson M.N., Faraday B.J., Hoover J.I. (1965). Room temperature oxidation of silicon during and after etching. J. Phys. Chem. Solids.

[41] Liao W.-S., Lee S.-C. (1996). Water-induced room-temperature oxidation of Si–H and –Si–Si– bonds in silicon oxide. J. Appl. Phys..

[42] Massoud H.Z. (1995). The onset of the thermal oxidation of silicon from room temperature to 1000 °C. Microelectron. Eng..

[43] Morita M., Ohmi T. (1994). Characterization and Control of Native Oxide on Silicon. Jpn. J. Appl. Phys..

[44] Taft E.A. (1988). Growth of Native Oxide on Silicon. J. Electrochem. Soc..

