# Supplementary material for: New Condensation Polymer Precursors Containing Consecutive Silicon Atoms—Decaisopropoxycyclopentasilane and Dodecaethoxyneopentasilane—And Their Sol–Gel Polymerization
Source: Polymers (Basel). 2019 May 9;11(5):841. doi: 10.3390/polym11050841 (PMC6572446; doi:10.3390/polym11050841)
Supplement: Supplementary file 1 [file polymers-11-00841-s001.pdf]

Supplementary Information

New Condensation Polymer Precursors Containing Consecutive Silicon Atoms, Decaisopropoxycyclopentasilane and Dodecaethoxyneopentasilane, and their Sol-gel Polymerization

Sung Jin Park[1], Myong Euy Lee[1], Hyeon Mo Cho[2,*] and Sangdeok Shim[3]

1 Department of Chemistry and Medical Chemistry, College of Science and Technology, Research and Education Center for Advanced Silicon Materials, Yonsei University, Wonju, Gangwon-do 26493, South Korea; melgg@yonsei.ac.kr
2 University College, Yonsei University, Incheon 21983, South Korea
3 Department of Chemistry, Sunchon National University, Sunchon 57922, South Korea; san90@scnu.ac.kr
* Correspondence: hyeonmo@yonsei.ac.kr

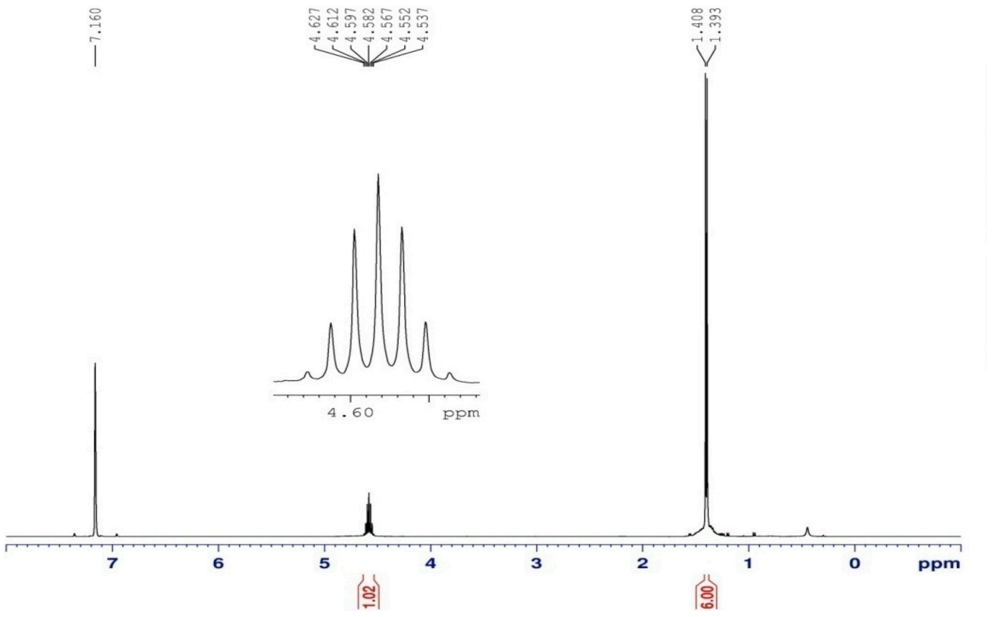

Figure S1. ^{1}H NMR spectrum of **CPS**.

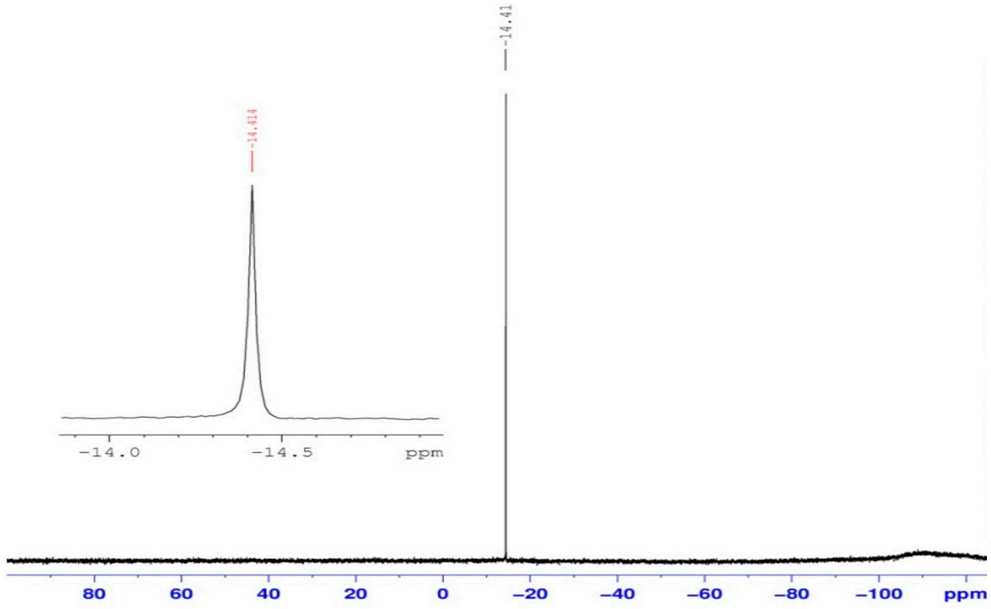

Figure S2. ^{29}Si NMR spectrum of **CPS**.

[Elemental Composition] Page: 1

Data : FAB-C315
Sample: Dipro
Note : m-NBA
Inlet : Direct Ion Mode : FAB+
RT : 0.33 min Scan#: (1,29)
Elements : C 100/0, H 150/0, O 10/0, Si 8/3
Mass Tolerance : 1000ppm, 3mmu if m/z < 3, 5mmu if m/z > 5
Unsaturation (U.S.) : 0.0 - 100.0

Observed m/z	Int%	Err[ppm / mmu]	U.S.	Composition
687.3267	40.7	-4.7 / -3.2	24.5	C 46 H 51 Si 3
		+4.4 / +3.0	6.5	C 31 H 59 O 9 Si 4
		-0.2 / -0.1	1.5	C 27 H 63 O 10 Si 5
		+3.8 / +2.6	5.5	C 30 H 63 O 6 Si 6
		-0.8 / -0.5	0.5	C 26 H 67 O 7 Si 7
		+3.2 / +2.2	4.5	C 29 H 67 O 3 Si 8

[Theoretical Ion Distribution] Page: 1

Molecular Formula : C27 H63 O10 Si5
(m/z 687.3268, MW 688.2187, U.S. 1.5)
Base Peak : 687.3268, Averaged MW : 688.2116(a), 688.2133(w)

m/z	INT.	
687.3268	100.0000	**
688.3284	55.7284	********************************
689.3269	33.5308	*******************
690.3275	12.0882	*******
691.3266	4.1137	**
692.3268	1.0807	*
693.3263	0.2596	
694.3266	0.0525	
695.3265	0.0096	
696.3270	0.0015	
697.3274	0.0002	

Figure S3. HRMS result of **CPS**.

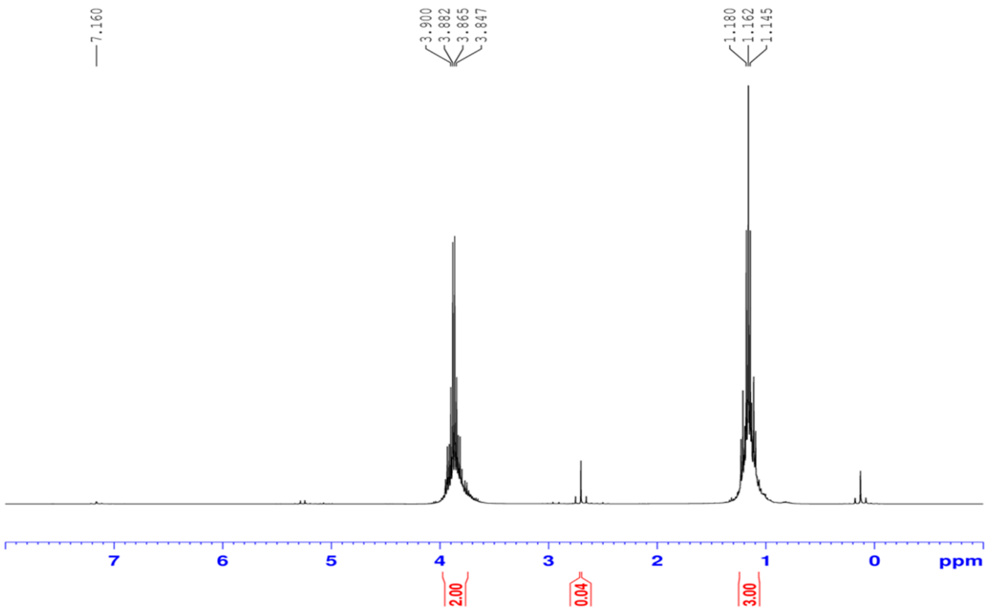

Figure S4. ^{1}H NMR spectrum of **NPS**.

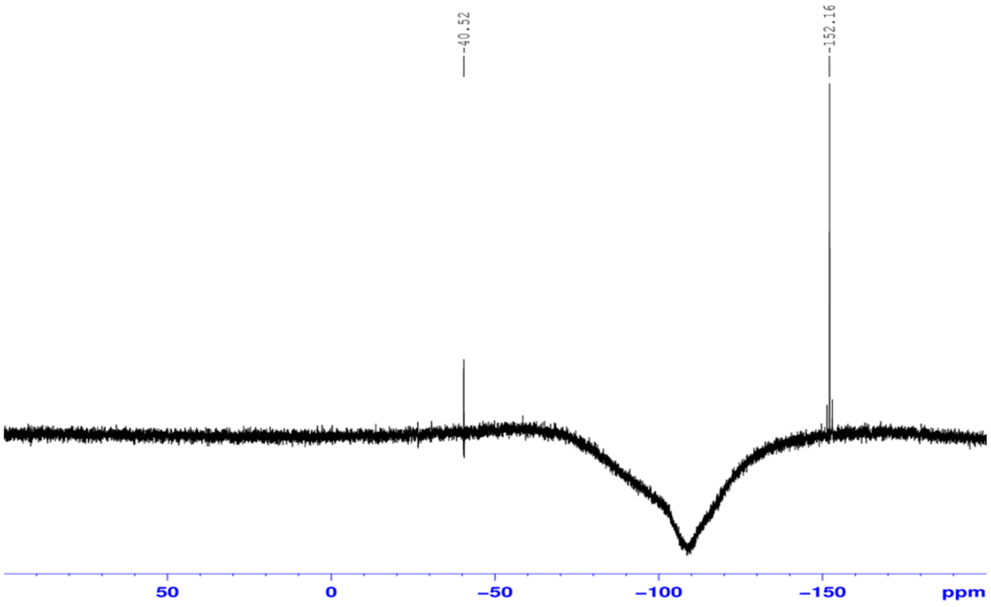

Figure S5. ^{29}Si NMR spectrum of **NPS**.

```
[ Elemental Composition ]                                              Page: 1
Data   : FAB-H757                      Date : 24-Apr-2014 16:50
Sample: NPSOEt
Note : m-NBA
Inlet : Direct                         Ion Mode : FAB+
RT : 0.87 min                          Scan#: (37,39)
Elements : C 150/0, H 150/0, O 15/10, Si 6/4
Mass Tolerance      : 1000ppm, 3mmu if m/z < 3, 5mmu if m/z > 5
Unsaturation (U.S.) : -10.0 - 100.0

Observed m/z  Int%   Err[ppm / mmu]    U.S.  Composition
  681.3030     9.5      +3.0 /  +2.0   -0.5  C 24 H 61 O 12 Si 5
                        -1.6 /  -1.1   -5.5  C 20 H 65 O 13 Si 6


[ Theoretical Ion Distribution ]                                       Page: 1
Molecular Formula : C24 H61 O12 Si5
                       (m/z   681.3009, MW   682.1686, U.S. -0.5)
Base Peak :   681.3009, Averaged MW :   682.1614(a),   682.1631(w)

   m/z       INT.
  681.3009 100.0000 **********************************************************
  682.3025  52.4679 ******************************
  683.3009  32.1864 *******************
  684.3015  11.2438 *******
  685.3005   3.8707 **
  686.3008   0.9992 *
  687.3003   0.2422
  688.3006   0.0486
  689.3006   0.0090
  690.3011   0.0014
  691.3015   0.0002
```

Figure S6. HRMS result of **NPS**.

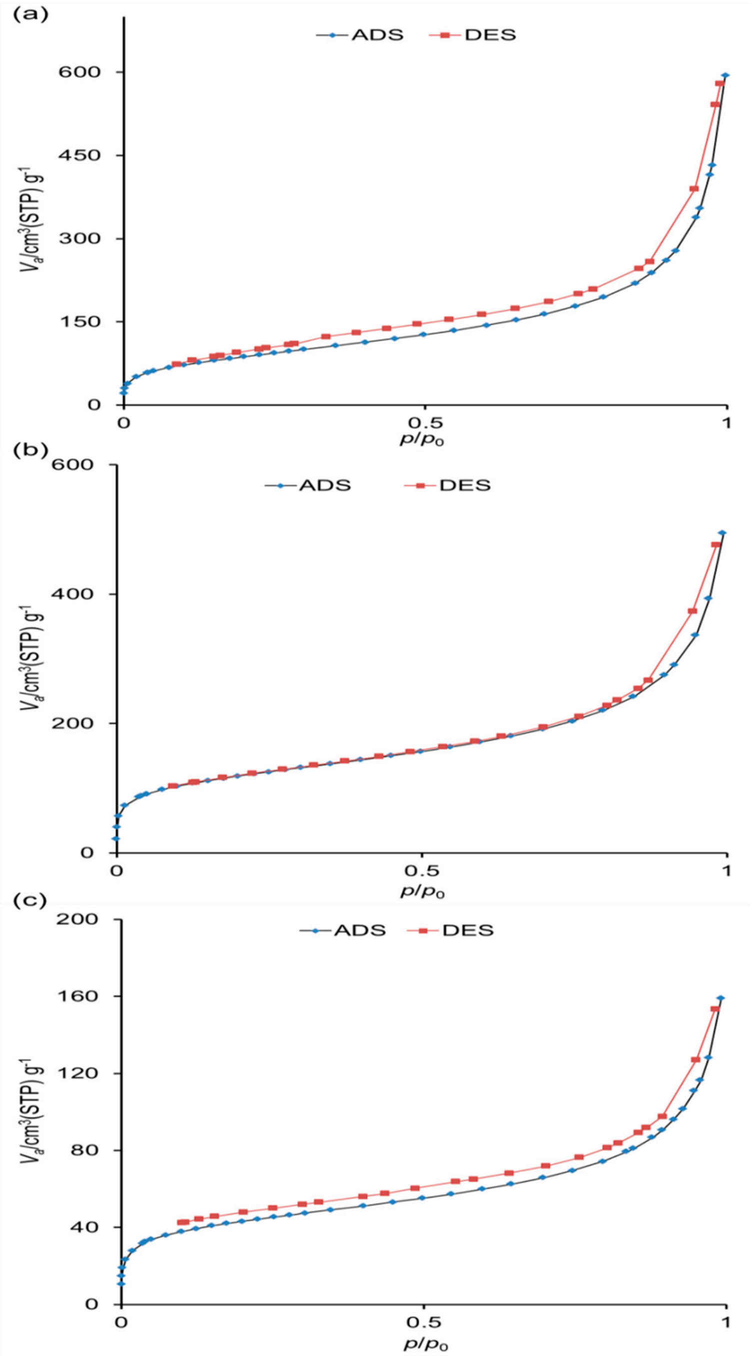

Figure S7. N_2 adsorption-desorption isotherms of the **CPS xerogel**s. (a) **CPSH**, (b) **CPSOH**, (c) **CPSN.**

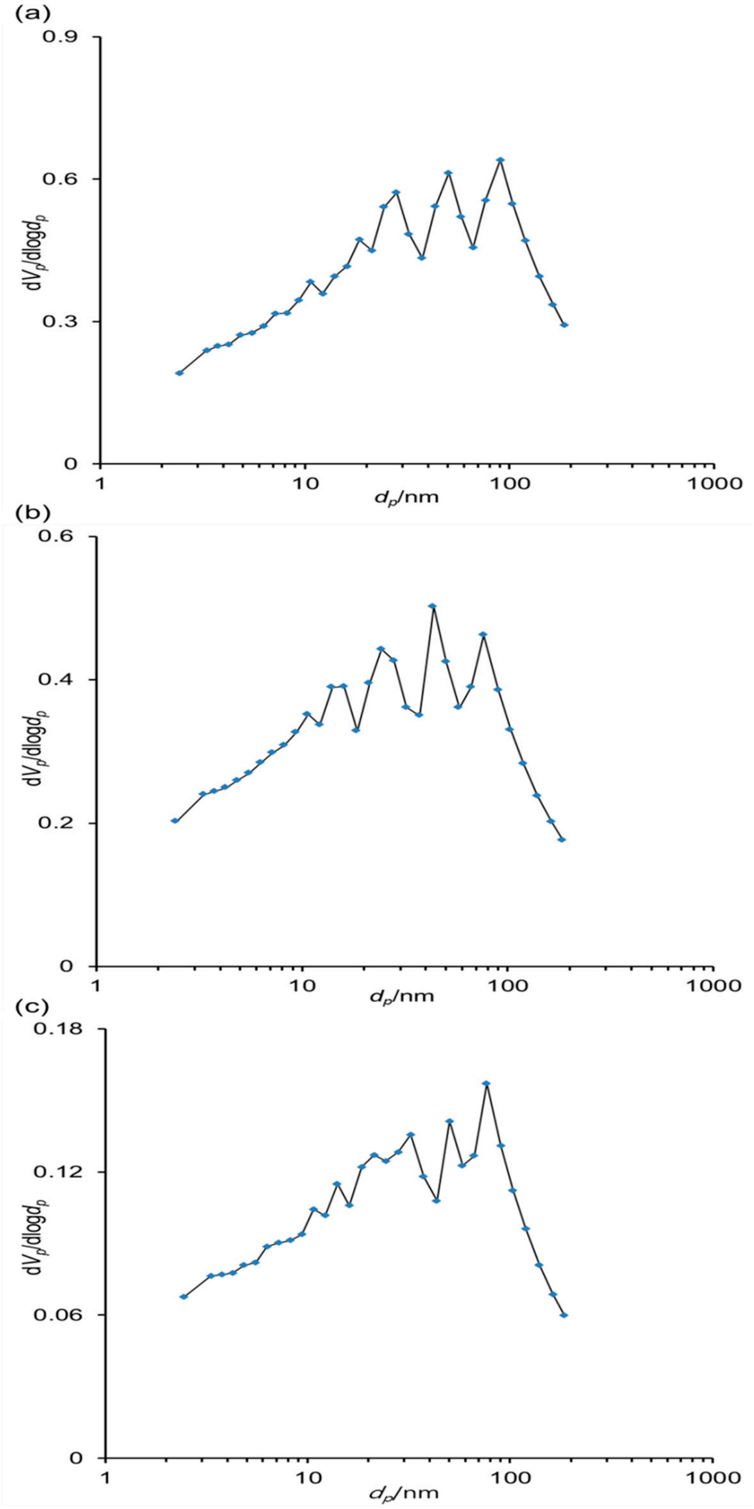

Figure S8. Pore size distribution of the **CPS xerogel**s. (a) **CPSH**, (b) **CPSOH**, (c) **CPSN.**
